# The bibliometric and visualized analysis on cancer stem cells in the early 21st century

**DOI:** 10.1097/JS9.0000000000003368

**Published:** 2025-09-20

**Authors:** Ludan Zhang, Fanyu Meng, Rui Ge, Xin Tian, Peng Sun, Zhongqing Wang, Xiaojing Yan

**Affiliations:** a Department of Hematology, The First Hospital of China Medical University, Shenyang, Liaoning, China; b Department of Information Center, The First Hospital of China Medical University, Shenyang, Liaoning, China; c Department of Ophthalmology, The First Hospital of China Medical University, Shenyang, Liaoning, China

**Keywords:** bibliometrics, cancer stem cells, CiteSpace, drug resistance, VOSviewer

## Abstract

Cancer stem cells (CSCs) are a crucial tumor subpopulation, involved in tumor initiation, metastasis and recurrence due to their unique abilities for self-renewal and differentiation. This bibliometric analysis aims to illustrate the current landscape of CSC research from 2001 to 2024, using data from the Web of Science Core Collection. We examined 20 839 publications, analyzing trends in annual publications, author contributions, institutional contributions and keyword co-occurrence. Our findings revealed a rising trend in research output, with the United States and China leading in publication volume. The top two institutions were Sun Yat-sen University and the University of Texas MD Anderson Cancer Center, with Harvard University having the highest average citations. Furthermore, keyword analysis identified major thematic clusters, including (1) Cluster 1 focused on the CSCs biomarkers and drug resistance. (2) Cluster 2 focused on the metabolism of CSCs, including oxidative stress, apoptosis, autophagy, the cell cycle and angiogenesis. (3) Cluster 3 focused on CSCs in terms of self-renewal, differentiation, and quiescence. (4) Cluster 4 emphasized the crucial role of CSCs in tumor metastasis, invasion, migration as well as their regulation pathways. (5) Cluster 5 focused on CSCs in immunotherapy and tumor microenvironment. This study enhances understanding of research trends and guides future efforts in targeting CSCs for therapeutic interventions, especially in surgical diagnosis and treatment. By illuminating key contributions and emerging themes, it serves as a valuable resource for researchers aiming to advance the field.

**HIGHLIGHTS**

- This study provides a thorough bibliometric and visualized analysis of cancer stem cell (CSC) research from 2001 to 2024, examining over 20 000 publications.
- The research reveals the rising trend in CSC studies, with the United States and China leading in publication volume and significant contributions from institutions like MD Anderson Cancer Center, Sun Yat-sen University, and Harvard University.
- Identified key thematic clusters include CSC biomarkers, metabolism, tumor metastasis, immunotherapy and drug resistance.
- The findings guide future efforts in targeting CSCs for therapeutic interventions, focusing on emerging topics like ferroptosis, organoids, and extracellular vesicles.

## Background

Cancer stem cells (CSCs) are a subpopulation of cancer capable of self-renewal and differentiation[1]. They were first identified in 1994 when researchers isolated them from leukemia stem cells (LSCs)[2]. Further researches indicated that CSCs are also present in other types of tumors, including hepatocellular cancer, breast cancer, and so on^[3–5]^. The infinite proliferation and differentiation capacity of CSCs, along with their quiescent state in the core of tumor masses, make them resistant to chemotherapy and radiotherapy^[6–8]^. Meanwhile, CSCs lead to tumor recurrence, as the remaining CSCs can quickly regenerate tumorigenic and highly invasive cells, reconstructing an aggressive tumor[9]. Therefore, focusing on the eradication of CSCs is a crucial strategy for reducing tumor resistance and recurrence. Over the last 20 years, publications have described various targeting strategies including: (1) Targeting classic markers of CSCs like CD13, CD44, CD133, Nanog, ALDH, and SOX2; (2) Targeting major pathways of CSCs like Wnt, Notch, Hh, NF-κB, JAK-STAT, TGF/SMAD, PI3K/AKT/mTOR, PPAR signaling pathway; (3) tumor microenvironment targeting strategies; (4) immune modification strategies; (5) agent-induced differentiation strategies^[10,11]^.

Bibliometric analysis is a method used to analyze academic publications quantitatively. It uses statistical tools, including keyword analysis, citation analysis, social network analysis and cluster analysis, to examine the impact, structure, and trends within scientific research[12]. In recent years, several bibliometric studies have examined the literature in clinical medicine and biomedicine. By bibliometric analysis, researchers can more effectively evaluate research impact and discover research trends, so as to direct the future research.

Currently, there are multiple articles and reviews on CSCs mechanisms, microenvironment and target therapy. But quantitative and qualitative analyses of CSCs were limited. With bibliometric analysis, the current state and trends of research fields in CSCs were first characterized. We collected publications from the Web of Science Core Collection (WoSCC) covering the period 2001–2024 and analyzed various metrics, including the distribution of annual publications, countries, institutions, authors, journals, keyword co-occurrence, and co-citation patterns. Moreover, we will discuss in-depth the impact of these key issues, aiming to assist researchers in understanding the scope of current research topics and discovering new fields on CSCs as well as provide a basis for CSC in surgical care. This work has been checked in line with the TITAN Guidelines (2025) for the declaration and use of artificial intelligence (AI) in research[13].

## Method

### Data source and search strategy

**Figure 1.**
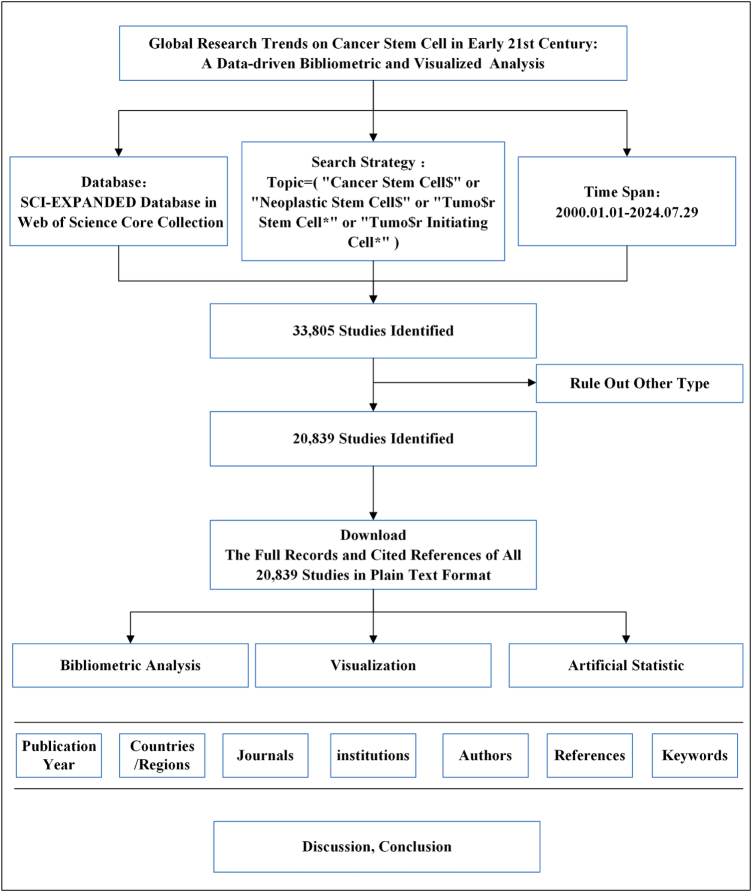
The data collection and retrieval strategy.

Web of Science is a professional and comprehensive citation database which provides extensive bibliometric information, such as citations and references. Therefore, Web of Science was selected as the data source for this bibliometric analysis. The search was conducted in the Science Citation Index Expanded Database within the WoSCC. The search strategy was as follows: Topic = (“Cancer Stem Cell$” or “Neoplastic Stem Cell$” or “Tumor Stem Cell*” or “Tumor Initiating Cell*”) (Fig. 1). Publications on the topic of CSCs between 1 January 2000 and 29 July 2024 were searched on the WoS core collection. The publication language was limited to English and the publication type was restricted to articles. To avoid the potential impact of database updates, all bibliometric data for 20 839 publications were downloaded on 29 July 2024. As the data came from public database, there was no need for ethics committee approval.

### Data analysis

This study primarily utilized VOSviewer (version 1.6.18) for the analysis of country/region, institution, author relationships, journal citations, and keyword co-occurrence. VOSviewer is a software tool designed for visualization and construction in bibliometrics[14]. It is a free, Java-based software developed by Van Eck and Waltman from the Centre for Science and Technology Studies (CWTS) at Leiden University, the Netherlands, in 2009[15]. The main functions of VOSviewer include network visualization, overlay visualization and density visualization, which are based on co-occurrence clustering and clustering visualization of impact units[16]. Along with knowledge map visualization, it also provides data cleaning and term filtering features. Additionally, CiteSpace software was used for burst analysis of references to complement the analysis.

## Results

### Number of annual global publications

**Figure 2.**
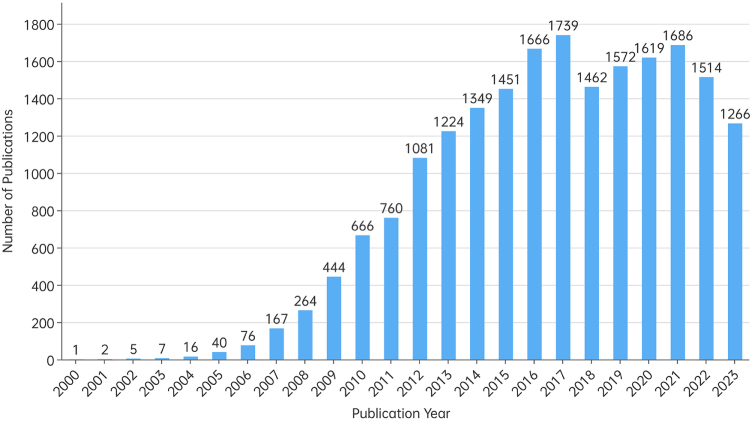
Annual global publications on cancer stem cells between 2000 and 2023.

The number of publications reflects the popularity and development trends of the CSCs field. The number of annual global publications from 2000 to 2023 is depicted in Figure 2 (data for the full year of 2024 are not included). The publications showed an overall upward trend, with the number of publications increasing from less than 10 between 2000 and 2003 to consistently exceeding 1000 in recent years. From 2000 to 2017, the number of publications increased rapidly, peaking at 1739 in 2017. Although there was a slight decline in 2018, the publication volume steadily increased again over the following 3 years. However, a slight downward trend has been observed from 2021 to 2023.

### Contributions of countries/regions

**Figure 3.**
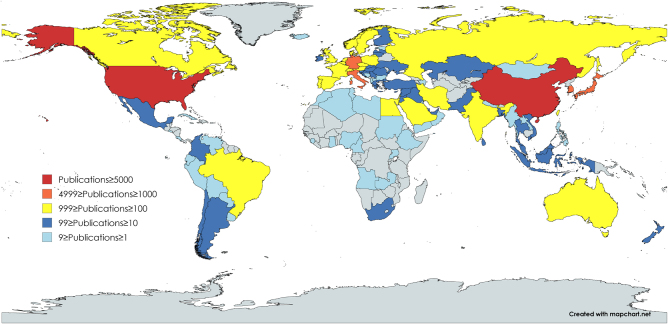
Global distribution of countries/regions.

A total of 109 countries and regions have published research articles on CSCs. Based on publication volume, these countries/regions were represented in different colors on the global distribution map (Fig. 3). Countries/regions with publication volumes of 5000 or more were represented in red; those with 1000–4999 publications were shown in orange; those with 100–999 publications were depicted in yellow; those with 10–99 publications were colored blue; and those with 1–9 publications were marked in cyan. The visualization results indicated that only the United States and China fell into the first step. Countries/regions in the second step were predominantly located in Europe, while those in the fifth step were mainly located in Africa and Southeast Asia.

The top 20 most productive countries in the field of CSCs are listed in Table 1. China (6863 publications, 217 144 citations) was the most productive country, followed by the USA (6313 publications, 425 337 citations), which these two countries account for more than half of the total publications in this field. Although the number of publications from Switzerland was ranked 16th, its average citations were the highest. Of the majority of countries/regions, the average publication year was between 2015 and 2019, which aligned with a notable increase in the volume of publications in this field. Among the top three countries by publication volume – China, the USA, and Japan – the average publication years are 2018.08, 2015.75, and 2016.38, respectively.

**Table 1:** Top 20 most productive countries/regions

Rank	Countries/Regions	Counts	Citations	Avg.Citations	Avg.Pub.Year
1	China	6863	217 144	32	2018.08
2	United States of America	6313	425 337	67	2015.75
3	Japan	1733	64 328	37	2016.38
4	Italy	1246	64 791	52	2016.46
5	Germany	1166	70 729	61	2016.10
6	South Korea	1051	34 882	33	2017.29
7	United Kingdom	863	49 511	57	2016.37
8	Taiwan (China)	749	29 679	40	2017.32
9	Canada	712	52 955	74	2015.71
10	India	656	16 136	25	2019.07
11	Spain	639	28 722	45	2017.18
12	France	579	32 000	55	2016.53
13	Australia	433	21 147	49	2016.85
14	Iran	406	7338	18	2019.21
15	Netherlands	310	24 188	78	2016.06
16	Switzerland	277	22 651	82	2016.16
17	Sweden	258	10 955	42	2016.91
18	Brazil	246	5596	23	2017.82
19	Poland	222	5359	24	2017.69
20	Egypt	200	3556	18	2019.43

### Contributions of institutions

**Figure 4.**
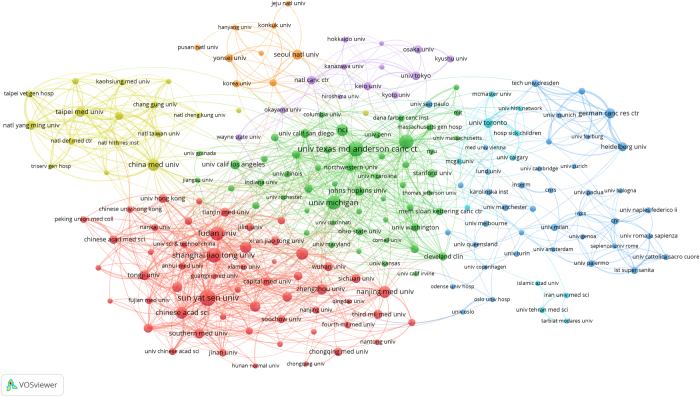
The collaboration network map of 198 institutions.

A total of 11 927 institutions have published researches on CSCs. After setting a minimum publication threshold of 50, we identified 198 institutions. The collaboration network of these 198 institutions is visualized in Figure 4. The largest cluster is the red cluster formed by 55 institutions centered around Fudan University, Shanghai Jiao Tong University, and Sun Yat-sen University. Additionally, the top three institutions with the most collaborators were Harvard University (*n* = 142), The University of Texas MD Anderson Cancer Center (*n* = 138), and the University of Michigan (*n* = 106).

The top 20 most productive institutions in the field of CSCs are listed in Table 2. The top three institutions were Sun Yat-sen University (499 publications, 22 949 citations), the University of Texas MD Anderson Cancer Center (443 publications, 37 567 citations), and Shanghai Jiao Tong University (424 publications, 10 572 citations). Additionally, Harvard University had the highest average citations (111 average citations), while Southern Medical University had the most recent average publication year (Average Publication Year: 2018.87).

**Table 2:** Top 20 most productive institutions

Rank	Institution	Counts	Citations	Avg.Citations	Avg.Pub.Year
1	Sun Yat-sen University	499	22 949	46	2017.85
2	The University of Texas MD Anderson Cancer Center	443	37 567	85	2015.01
3	Shanghai Jiao Tong University	424	15 072	36	2017.40
4	Fudan University	398	15 700	39	2017.59
5	Harvard University	389	43 366	111	2015.40
6	University of Michigan	363	37 697	104	2015.21
7	China Medical University	288	11 028	38	2018.05
8	Huazhong University of Science And Technology	277	10 328	37	2017.75
9	National Cancer Institute	273	21 429	78	2014.76
10	Chinese Academy of Sciences	268	12 930	48	2017.85
11	Nanjing Medical University	267	7005	26	2018.69
12	Zhejiang University	253	8766	35	2017.69
13	Peking University	218	7058	32	2017.71
14	Zhengzhou University	207	5022	24	2018.74
15	Tongji University	206	7551	37	2018.39
16	Seoul National University	205	6793	33	2016.44
17	Naval Medical University	198	9373	47	2016.68
18	German Cancer Research Center	196	8948	46	2016.73
19	Southern Medical University	196	5034	26	2018.87
20	Taipei Medical University	190	5179	27	2018.30

### Contributions of authors

A total of 102 288 authors have published in the field of CSCs. The top 20 most productive authors in this field are listed in Table 3. The author contributing to this field most was Max S. Wicha, with 96 publications, significantly outpacing other authors. The other two top authors by publication volume were Jeremy N. Rich (69 publications) and Justin D. Lathia (60 publications). Although Qiulian Wu ranks 16th in terms of publication volume within the top 20 list, he held the highest average citations (280 publication citations).

**Table 3:** Top 20 most productive authors

Rank	Author	Counts	Citations	Avg.Citations	Avg.Pub.Year
1	Wicha, Max S.	96	16 549	172	2014.10
2	Rich, Jeremy N.	69	15 554	225	2014.70
3	Lathia, Justin D.	60	6498	108	2015.80
4	De Maria, Ruggero	58	6103	105	2014.93
5	Mori, Masaki	56	2848	51	2014.70
6	Saya, Hideyuki	56	3122	56	2015.36
7	Stassi, Giorgio	50	9953	199	2015.22
8	Doki, Yuichiro	47	2214	47	2015.34
9	Yu, Cheng-Chia	46	2553	56	2016.15
10	Todaro, Matilde	44	7988	182	2014.98
11	Yeh, Chi-Tai	44	1241	28	2017.73
12	Lisanti, Michael P.	41	3094	75	2016.93
13	Dou, Jun	40	1383	35	2015.43
14	Kitanaka, Chifumi	40	1572	39	2016.55
15	Seno, Masaharu	39	837	21	2019.54
16	Wu, Qiulian	39	10 905	280	2015.79
17	Ma, Stephanie	38	5221	137	2015.68
18	Medema, Jan Paul	38	6499	171	2013.71
19	Okada, Masashi	36	1432	40	2017.33
20	Sotgia, Federica	36	2493	69	2017.25

### Contributions of journals

The top 20 productive journals in the field are listed in Table 4. The number of publications published by these journals accounted for 29.62% of all publications. The top three journals by publication volume are *Oncotarget* (818 publications, 32 217 citations), *PLOS One* (671 publications, 34 767 citations), and *Cancer Research* (432 publications, 56 673 citations). The *Proceedings of the National Academy of Sciences of the United States of America* led in average citations per article, significantly outpacing other journals, with an average of 187.96 citations. Most journals had an average publication year clustered between 2013 and 2018. The three most recent journals, based on average publication year, were *Cancers* (2020.88), *International Journal of Molecular Sciences* (2020.87), and *Frontiers in Oncology* (2020.88).

**Table 4:** Top 20 most productive journals

Rank	Journal	Counts	Citations	Avg.Citations	Avg.Pub.Year
1	*Oncotarget*	818	32 217	39.39	2015.79
2	*PLOS One*	671	34 767	51.81	2013.86
3	*Cancer Research*	432	56 673	131.19	2014.23
4	*Cancers*	426	6005	14.10	2020.88
5	*Scientific Reports*	392	12 029	30.69	2018.23
6	*International Journal of Molecular Sciences*	377	4638	12.30	2020.87
7	*Oncogene*	355	26 709	75.24	2016.21
8	*Cancer Letters*	302	13 401	44.37	2016.79
9	*Oncology Reports*	287	7245	25.24	2015.45
10	*BMC Cancer*	281	9604	34.18	2016.44
11	*Cell Death & Disease*	258	10 499	40.69	2018.67
12	*Oncology Letters*	258	3649	14.14	2017.04
13	*International Journal of Oncology*	237	8170	34.47	2014.92
14	*Biochemical and Biophysical Research Communications*	228	7218	31.66	2015.46
15	*Frontiers in Oncology*	215	2876	13.38	2020.88
16	*Stem Cells*	213	19 730	92.63	2013.11
17	*Clinical Cancer Research*	183	20 046	109.54	2014.33
18	*Anticancer Research*	167	2852	17.08	2017.07
19	*Cell Cycle*	165	9501	57.58	2013.19
20	*Proceedings of the National Academy of Sciences of the United States of America*	165	31 014	187.96	2013.28

**Figure 5.**
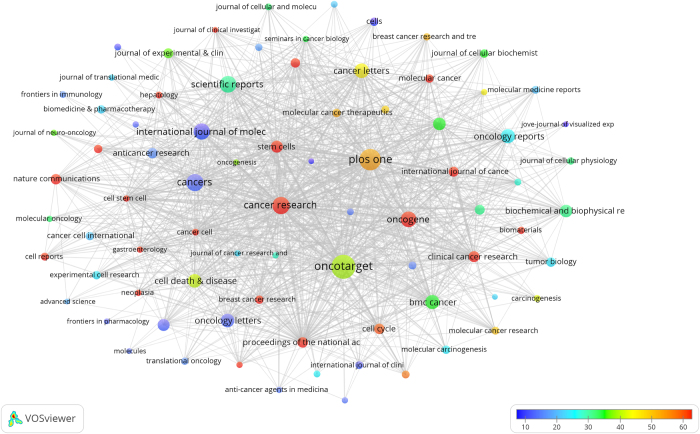
The overlay visualization map of 80 productive journals.

A total of 1697 journals have contributed to the field of CSCs research. By setting a minimum publication threshold of 50, we identified 80 productive journals. Figure 5 presents an overlay visualization of the average citation counts for these journals. In this figure, the node size represented the publication volume of each journal, while the node color indicated the average citation count. Notably, 19 journals, including *Cancer Research, Stem Cells, Clinical Cancer Research*, and *Proceedings of the National Academy of Sciences of the United States of America*, had an average citation count exceeding 60, highlighting their significant contribution of high-quality research to this field.

### Analysis of references burst detection

**Figure 6.**
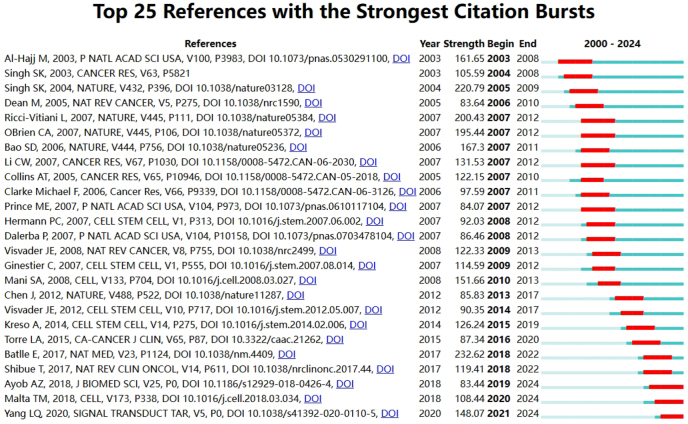
Visualization map of top 25 references with the strongest citation bursts on CSCs.

This study included a total of 20 839 research articles. Using the burst analysis of references in CiteSpace, we identified the 25 most influential references in this field (Fig. 6 and Supplementary Digital Content Table S2, available at: http://links.lww.com/JS9/F129). It is evident that most burst publications experienced citation surges either in the year of publication or the following year. Citation bursts in this study began in 2003, marked by Batlle, Eduard. Citation bursts have continued in subsequent years, with the most recent burst starting with a 2020 publication by Yang LQ, which began gaining traction in 2021 and persists to the present.

### Co-occurrence analysis of author keywords

**Figure 7.**
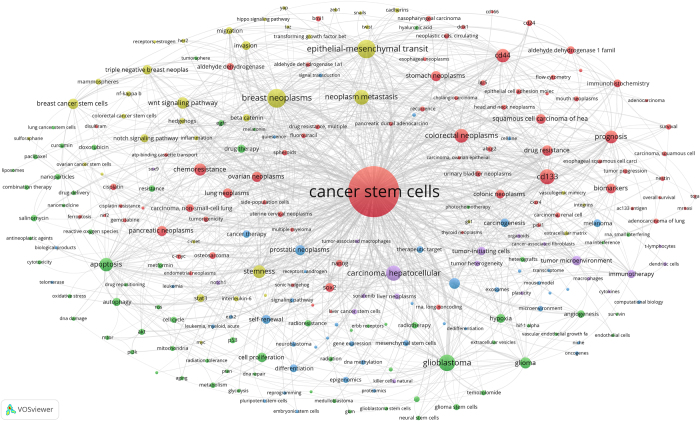
The co-occurrence network map of author keywords on CSCs.

The 20 839 publications on CSCs contained a total of 21 399 author keywords. As shown in Figure 7 and Supplementary Digital Content Table S1, available at: http://links.lww.com/JS9/F128, we set the threshold for high-frequency keywords at 30, resulting in 247 high-frequency author keywords. These keywords were then subjected to a co-occurrence analysis, leading to the construction of a co-occurrence network map of high-frequency keywords. The co-occurrence network map of high-frequency keywords was composed of five distinct clusters, each represented by a different color. The largest cluster (red cluster), with 78 keywords, focused on the CSCs biomarkers and drug resistance. Cluster 2 (green cluster) focused on CSCs including oxidative stress, apoptosis, autophagy, the cell cycle, and angiogenesis. Cluster 3 (blue cluster) focused on CSCs in terms of self-renewal, differentiation, and quiescence. Cluster 4 (yellow cluster) focused on the crucial role of CSCs in tumor metastasis, invasion, migration and their regulation pathways. Cluster 5 (purple cluster) focused on CSCs in immunotherapy and tumor microenvironment.

**Figure 8.**
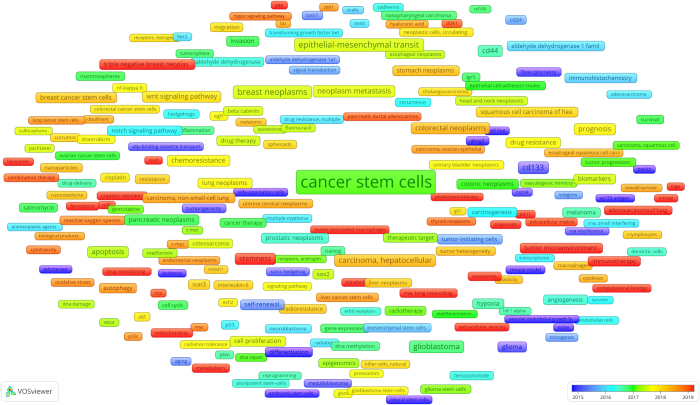
The overlay visualization map of author keywords on CSCs.

We utilized VOSviewer to construct an overlay visualization of the 247 high-frequency author keywords, as shown in Figure 8. Each node’s color represented its average publication year, ranging from blue to red, with blue indicating earlier years and red indicating more recent publication years. Over the past 5 years, CSCs related researches have primarily focused on ferroptosis, The Cancer Genome Atlas (TCGA), organoids, and extracellular vesicles (EVs).

## Discussion

### Total trend and international cooperation

CSCs research has surged in recent years, with a steady rise in publications from 2000 to 2024. The period from 2000 to 2017 saw an exponential growth in articles, peaking at 1739 in 2017. In total 109 countries have published articles related to CSCs, with China and the United States leading the way, each with over 5000 publications. Notably, Switzerland held the top position for average citations per article, indicating a higher overall quality of its publications. The collaboration networks, largely reflecting national trends, were primarily centered in China and the United States, with the top three contributing institutions being Sun Yat-sen University, The University of Texas MD Anderson Cancer Center, and Shanghai Jiao Tong University. Among these, Harvard University stood out with the highest average citation per publication. This clearly demonstrates the leading position of China and the United States in CSC research.

In this field, Qiulian Wu, with the highest average citation count, focused on glioma stem cells and their tumor microenvironment. Her groundbreaking discovery that “Glioma stem cells promote radioresistance by preferential activation of the DNA damage response” has been widely cited[17]. A notable burst occurred with a 2017 review by Eduard Batlle, summarizing advancements in CSC research and insights into normal stem cell functions in tissue maintenance[1]. The most recent significant citation burst was for Yang LQ’s 2020 review on CSC pathway targeting for cancer therapy, reflecting the growing emphasis on CSC-focused therapeutics[11].

### Research hotspots

The largest cluster (red cluster), comprising 78 keywords, focused on the CSCs biomarkers and drug resistance. CSC biomarkers are categorized into cell-surface markers and intracellular markers, with CD133 and CD44 being key cell surface glycoproteins that serve as vital CSC biomarkers across various cancers[18]. CD133 and CD44 serve as critical CSC biomarkers in surgical oncology, enabling early diagnosis through liquid biopsy, such as exosomal detection in blood/CSF, prognostication of recurrence risk, and guidance of surgical decisions. Concurrently, nanoparticle-targeted delivery systems (liposome-encapsulated chemotherapeutics) directed at CD133^+^ cells are under investigation in GBM, aiming to eliminate residual CSCs post-resection. Furthermore, CD133 silencing enhances radiosensitivity in GBM cells, providing a novel therapeutic target for adjuvant post-operative radiotherapy^[19,20]^.

Another significant intracellular biomarker is Sox2, which plays a crucial role in maintaining an undifferentiated cellular phenotype[21]. Its abnormal expression in various cancers often leads to increased chemotherapy resistance and promotes asymmetric cell divisions, as observed in colorectal cancer[22]. Aldehyde dehydrogenase (ALDH), particularly ALDH1, is a surface marker in maintenance of stemness and tumorigenicity of cancer cells, with its family playing central roles in downstream transcriptional regulation across Wnt/βcatenin, JAK/STAT, hedgehog pathways, and so on^[23,24]^. Consequently, its expression is associated with enhanced tumorigenic potential and resistance to therapies, suggesting targeting ALDH1 as a therapeutic strategy. In Cluster 1, another significant keyword was drug resistance, due to high expression of multidrug resistance transporters in CSCs, enhanced DNA repair mechanisms or the ability to enter a quiescent state^[6,25]^. CSC markers, such as ALDH and CD133, confer resistance to selected anticancer drugs through metabolic inactivation, potentially contributing to cancer relapse^[26–28]^. These markers play crucial roles in cell adhesion, migration, tumorigenesis, and metastasis^[29–31]^.

Cluster 2 (green cluster) focused on the metabolism of CSCs, involving various crucial processes, including oxidative stress, apoptosis, autophagy, cell cycle, and angiogenesis. Tumor angiogenesis is essential for tumor growth and metastasis. Tumor angiogenesis also provides the oxygen and nutrients necessary for the survival and proliferation of CSCs. While angiogenesis inhibitors initially showed potential for solid tumors, clinical efficacy proved limited[32]. This prompted a shift toward vascular normalization therapy, aiming to remodel vessels into mature vessels for improved chemotherapy.

Autophagy is integral to ECs differentiation from CSCs and tumor angiogenesis[33]. Autophagy influences tumorigenesis and angiogenesis by inducing JAK2/STAT3 activation and affects tumor cell migration and invasion by regulating the secretion of pro-migratory cytokines and focal adhesion transformation^[34,35]^.

Autophagy is also regulated by reactive oxygen species (ROS) through protein modifications, including Beclin, Atg4, and Atg5. Additionally, indirect ROS regulators such as p38 and JNK can stimulate autophagy and inhibiting Akt signaling or downstream mTOR can further stimulate autophagy[36]. Elevated levels of ROS enhance autophagy by inhibiting glucose-6-phosphate dehydrogenase (G6PD) and inactivating the pentose phosphate pathway (PPP)^[37,38]^. Beyond its role in autophagy, oxidative stress can also influence CSCs through other pathways. The impact of ROS on mitochondrial function has consistently been a key area of research in CSCs. Conversely, photodynamic therapy (PDT) consumes a significant amount of oxygen by mediating the production of ROS, leading to mitochondrial hypoxia, which suppresses the stemness of cancer cells^[39–41]^. Additionally, ROS generated by PDT may disrupt CSC stemness by regulating cellular quiescence through lysosomal pathways, thereby exerting a therapeutic effect[42].

CSCs have a unique relationship with the cell cycle that distinguishes them from other cancer cells. CSCs often exhibit slower proliferation rates and longer quiescent phases, contributing to their resilience against therapies. The regulation of the cell cycle, involving proteins like cyclins and kinases, plays a crucial role in maintaining CSC self-renewal and differentiation. This interplay suggests that targeting cell cycle pathways could be a promising strategy for eradicating CSCs and improving cancer treatment efficacy[43].

Cluster 3 (blue cluster) focused on CSCs in terms of self-renewal, differentiation, and quiescence which are key properties influencing tumor recurrence and resistance. Jamieson et al. identified the self-renewal pathway as crucial for maintaining LSCs heterogeneity, with aberrant activation of the Wnt/β-catenin self-renewal pathway being a driving force in the propagation of human blast crisis LSCs^[44,45]^. Commonly, increased Wnt/β-catenin signaling has also been discovered in the maintenance of breast CSCs[46]. Additionally, multiple dysregulation in self-renewal pathways are functioning to maintain the CSC subset, such as PTEN, Wnt, hedgehog (Hh) signaling pathway, prompting numerous early-phase clinical trials focused on eliminating CSCs^[47–49]^. Another characteristic of CSCs is their ability to differentiate into various types of cancer cells. This multipotency is crucial for tumors growth and therapy resistance[50]. Therefore, dedifferentiating CSCs represents a potential strategy to enhance the efficacy of chemotherapy. Clinically, the use of all-trans retinoic acid for the treatment of acute promyelocytic leukemia is a successful strategy for differentiating CSCs[51]. Consequently, differentiation therapy has been explored as a strategy to suppress tumorigenesis by transforming highly malignant undifferentiated cancer cells into less tumorigenic, differentiated ones[52]. CSCs also exhibit cell quiescence, a reversible G0 phase that allows for rapid reentry into the cell cycle upon physiological stimuli, regulated by signaling pathways[53]. Cell quiescence enables CSCs resistance to therapy and tumor relapse, influenced by factors such as hypoxia, the CSC niche, and the extracellular matrix within the tumor microenvironment[8].

Cluster 4 (yellow cluster) focused on the crucial role of epithelial to mesenchymal transition (EMT) in tumor metastasis, invasion, migration, and their regulation pathways. The EMT and its reverse process, the mesenchymal-epithelial transition (MET), are essential for embryonic development and tissue repair. EMT confers malignant traits to carcinoma cells, including tumor metastasis, invasion, and migration. EMT equips tumor cells with increased stemness and heightened resistance to immune clearance and various therapeutic interventions[54]. In addition to its role in normal development, EMT is frequently aberrantly activated. Various signaling proteins play critical roles in driving EMT at different stages. The promotion of EMT requires the loss of E-cadherin expression, which is mediated by transcription factors such as SNAI1, TWIST1, ZEB1, ZEB2, and Slug^[55,56]^. Critical genes involved in cancer-related EMT are regulated by signaling pathways such as TGF, BMP, WNT, and NOTCH in various cancers, including colorectal cancer, triple-negative breast cancer, and lung cancer^[57–60]^. Thus, the interaction between EMT and CSC properties is vital for the migration and invasion of cancer cells during the metastatic process.

Cluster 5 (purple cluster) focused on CSCs in immunotherapy and tumor microenvironment. Immunotherapy strategies are novel therapeutic approaches that demonstrate promising results in targeting CSCs^[61,62]^. Researchers have examined CSCs’ immune properties and various approaches to target them, including monoclonal antibodies (mAbs), tumor vaccines, chimeric antigen receptor T (CAR-T) cells[63]. Recently, several mAbs and their constituents targeting CSC proteins have shown effectiveness against multiple tumors in clinical studies[64]. Reports indicated that anti-CD3/anti-CD133 bispecific antibodies demonstrate significant anti-tumor efficacy[65]. Catumaxomab has been shown to eliminate CD133+/EpCAM+ CSCs in cancers^[66–68]^. Another approach to targeting CSCs through immunotherapy involves tumor vaccines. Dendritic cell (DC) vaccines are based on the potent antigen-presenting function of DCs within the human immune system. DC vaccines can activate the patient’s own immune system to precisely target and attack cancer cells, applied to multiple clinical trials[63]. CAR-T immunotherapy has shown initial success in treating hematologic malignancies by reprogramming T lymphocytes to target specific antigens[69]. Evidence suggests CAR-T cells can effectively target CSCs in solid tumors like melanoma, glioblastoma, and breast cancer by focusing on associated antigens^[70,71]^.

Similar to normal stem cells, CSCs require a microenvironment to sustain their growth and self-renewal functions. The composition of the tumor microenvironment (TME) includes various cellular and noncellular components that interact with CSCs, influencing their self-renewal and differentiation. Key components include cancer-associated fibroblasts (CAFs), endothelial cells, adhesion molecules, signaling molecules, and extracellular matrix[72]. CSCs do not passively respond to the microenvironment but actively remodel it to support tumor progression. Christina Scheel et al. described three signaling pathways – TGF-β, canonical Wnt, and noncanonical Wnt – that work together to activate the EMT program and subsequently function in an autocrine manner to sustain the resulting mesenchymal state. Conversely, disrupting autocrine signaling by introducing inhibitors of these pathways could reduce tumorigenicity and metastasis[73].

The ability to evade the immune system and initiate tumors even under immune surveillance is another key characteristic of CSCs[74]. CSCs may secrete immunosuppressive factors and recruit immunosuppressive noncancerous cells. Subsequently, the immune suppressive cells, in turn, induce and maintain CSCs. Studies showed that some CSCs reduced MHC expression and hindered cytotoxic T-cell (CTL) recognition and immune response, suggesting the mechanism of immune evasion[75]. In head and neck cancer, CD44+ CSCs downregulate MHC and promote immunosuppressive pathways, while IFNγ can restore some CTL sensitivity^[76,77]^. A variety of humoral factors are involved in regulating the immune response, and this also applies to immune evasion by CSCs. In breast and glioma CSCs, TGFβ activation enhances stemness and immune evasion based on downregulation MHC and NKG2D and inducing Tregs[78]. Additionally, IL-6 is vital in CSC induction, particularly in lung, breast, and prostate cancers, driving STAT3 activation and promoting immune suppression through PD-L1 expression and MDSC recruitment^[3,79]^. CCL20, secreted by CSCs, reinforces immune evasion by recruiting Tregs through the CCR6 axis, further promoting immune resistance, tumor progression, and metastasis in hepatocellular carcinoma and breast cancer^[80,81]^. Hypoxia is recognized as a prominent characteristic of the TME and is believed to play a significant role in promoting the CSCs phenotype and increasing tumorigenicity[82]. Hypoxia-inducible factors (HIFs), especially HIF-1α and HIF-2α, regulate pathways like Akt, mTOR, Notch, and TGF-β, driving CSC self-renewal and inhibiting differentiation^[83,84]^. The relationship between CSCs and the TME is highly dynamic and complex. Understanding this interplay offers opportunities to develop more effective therapies that not only target the tumor but also disrupt the supportive environment that sustains CSCs.

### Emerging keywords on CSCs

Building on the analysis of existing research, new emerging keywords have started to gain prominence. These emerging topics not only reflect the evolving direction of the field but also provide valuable insights for future research. Building on the analysis of existing research, ferroptosis, TCGA, organoids, and EVs have emerged as the most prominent research hotspots in the past 5 years.

Ferroptosis, a novel form of programmed cell death, has gained considerable attention in cancer research, particularly for its potential in targeting CSCs[85]. Various pathways are involved in regulating ferroptosis in CSCs, including lipid metabolism, iron metabolism, NRF2 signaling, CD44 expression, Hippo-YAP/TAZ signaling, and autophagy. These mechanisms can selectively induce the death of CSCs[86]. For instance, SREBP-1 – a key transcriptional regulator of lipid synthesis – promotes hepatocellular carcinoma (HCC) progression by modulating fatty acid synthesis and cholesterol metabolism to influence tumor cell susceptibility to ferroptosis[87]. Similarly, the KEAP1-NRF2 axis protects tumor cells from ferroptosis by regulating oxidative stress and metabolic reprogramming, though its mutation confers resistance to chemotherapy and immunotherapy in lung cancer[88]. These findings reveal a bidirectional regulatory relationship between metabolic reprogramming and ferroptosis, offering novel therapeutic avenues. Ferroptosis in surgical oncology faces challenges including incomplete CSC eradication and ischemia-reperfusion-induced resistance[89]. A novel hydrogel eliminates residual CSCs by suppressing SLC7A11/GPX4/MMP7 to induce ferroptosis, while the Ce6-Erastin nanodrug synergizes with PDT through ferroptosis-driven ROS accumulation, oxygen elevation, and SLC7A11 inhibition to enhance cancer cell killing^[90,91]^.

In this context, the TCGA database provides a wealth of cancer genomic data that helps researchers identify key genes and molecular pathways related to CSCs, forming the foundation for drug screening and mechanistic studies. Analysis of the TCGA-BLCA cohort identified a 16-gene CSC prognostic signature (including FGFR2/ROBO2) predicting patient survival, where high-risk groups show significantly increased postoperative recurrence, suggesting the need for wider surgical resection or combined targeted therapy[92]. In lung adenocarcinoma (LUAD), integration of TCGA-LUAD and GEO data revealed CSC markers (e.g., MS4A2, IGSF10) that promote metastasis via MET, indicating that intraoperative frozen section assessment of resection margins is critical to address residual CSCs[93]. Collectively, TCGA-driven CSC research is transforming surgical oncology from “anatomic resection” to “molecular-functional resection,” with deeper studies of CSC-microenvironment, real-time intraoperative navigation, and combined surgery and CSC-targeting strategies to achieve curative outcomes.

Organoids, which are three-dimensional cell clusters derived from mammalian pluripotent stem cells or adult stem cells, are increasingly being used to model *in vivo* biological processes for research in tumor biology, pharmacology, and development. As a result, organoids have rapidly become one of the most prominent research areas today[94]. These emerging trends not only highlight the evolving direction of the field but also open up new avenues for future research.

EVs play a pivotal role in the communication between CSCs and TME, which can transport various chemicals, proteins, lipids, DNA and RNA fractions influencing tumor growth, angiogenesis, immune evasion, and cancer progression^[95,96]^. CSC-derived EVs can enhance stem-like properties in cancer cells, promote angiogenesis through pathways like miR-21/VEGF, and modulate the cancer progression by various miRNA upregulation^[97,98]^. They also interact with immune cells to regulate the host’s immune response, potentially inducing T cell apoptosis, suppressing NK cell activity and promoting the induction of regulatory T cells[99]. Furthermore, these exosomes contribute to drug resistance and tumor metastasis by transferring microRNAs that promote EMT and increase metastasis mediators^[98,100]^. Overall, the interplay between EVs and CSCs plays a significant role in shaping the tumor immune microenvironment, offering new insights for cancer therapy and potential biomarkers. Emerging hotspots and technologies are advancing CSC research and surgical applications: spatial transcriptomics, patient-derived CSC organoids enable personalized drug screening, and rapid intraoperative detection of CSC markers can refine resection boundaries in a short time.

### Limitations

As with other bibliometric studies, this research also has certain limitations. First, this study sourced publications on CSCs exclusively from the WoSCC database which is the most widely used and authoritative comprehensive database. A small part of non-English publications may be lost. Second, differences in institutional and author affiliations may result in discrepancies in the statistical outcomes for institutions and authors.

## Conclusion

The field of CSCs has experienced rapid growth since the early 21st century. Over the past 24 years, the number of publications has steadily increased. The United States and China have emerged as leading contributors to this field. Notably, four major research themes have been identified through keyword analysis: ferroptosis, TCGA, organoids, and EVs, which represent the current research hotspots. Collectively, these advances are redefining surgical oncology by shifting from traditional anatomic excision to “molecular-functional resection” where intraoperative CSC diagnostics, real-time margin assessment, and postoperative therapies synergize to reduce recurrence. In future, we need more dynamic CSC tracking technologies and closed-loop “resection-to-targeting” systems to achieve curative outcomes.

## Supplementary Material

## Data Availability

The data that support the findings of this study are available on request from the corresponding author.
